# Development of Nanozymatic Characteristics in Metal-Doped Oxide Nanomaterials

**DOI:** 10.1021/acs.jpcb.4c02526

**Published:** 2024-08-09

**Authors:** Julia Matysik, Olga Długosz, Marcin Banach

**Affiliations:** Faculty of Chemical Engineering and Technology, Institute of Chemistry and Inorganic Technology, Cracow University of Technology, Warszawska St. 24, Cracow 31-155, Poland

## Abstract

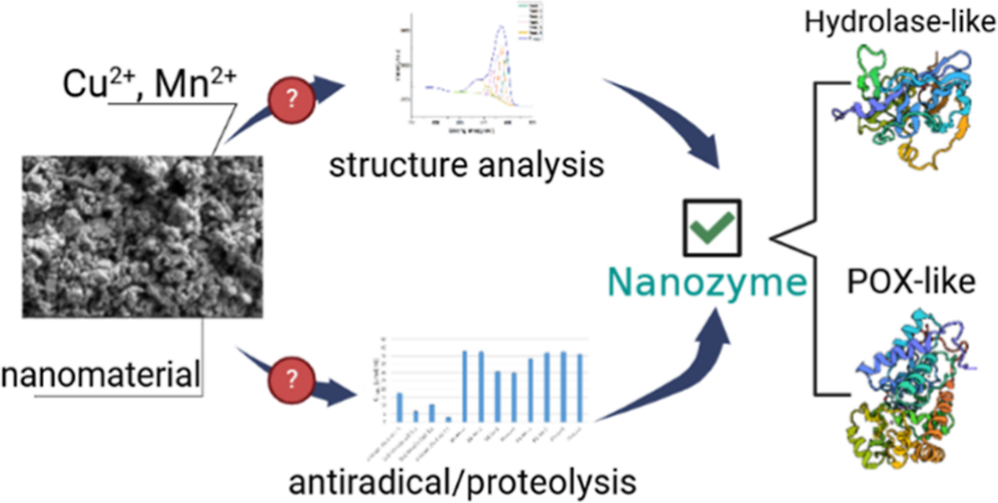

Nanozymes are nanoscale materials that exhibit enzymatic-like activity, combining the benefits of nanomaterials with biocatalytic effects. The addition of metals to nanomaterials can enhance their nanozyme activity by mimicking the active sites of enzymes, providing structural support and promoting redox activity. In this study, nanostructured oxide and silicate–phosphate nanomaterials with varying manganese and copper additions were characterized. The objective was to assess the influence of metal modifications (Mn and Cu) on the acquisition of the nanozymatic activity by selected nanomaterials. An increase in manganese content in each material enhanced proteolytic activity (from 20 to 40 mUnit/mg for BG-Mn), while higher copper addition in glassy materials increased activity by 40%. Glassy materials exhibited approximately twice the 2,2′-azino-bis(3-ethylbenzothiazoline-6-sulfonic acid radical activity (around 40 μmol/mL) compared to that of oxide materials. The proteolytic and antioxidant activities discussed in the study can be considered indicators for evaluating the enzymatic properties of the nanomaterials. Observations conducted on nanomaterials may aid in the development of materials with enhanced catalytic efficiency and a wide range of applications.

## 1 Introduction

Nanozymes, defined as nanometric materials exhibiting enzyme-like activity, have the potential to combine the advantages of nanomaterials with the catalytic effects provided by enzymes. Their formation processes are controlled, allowing for the creation of materials resembling enzymes and performing similar reactions.^1,2^ Natural enzymes exhibit limited thermostability and sensitivity to pH changes, and exceeding certain parameters may lead to irreversible changes in their structures. These environmental limitations do not apply to nanozymes, which can maintain enzymatic activity even under variable and unfavorable operational conditions.^3^ The preparation of nanomaterials plays a crucial role in successfully designing a matrix to utilize enzyme-like properties. Careful selection and synthesis of nanomaterials with desired properties are necessary to ensure subsequent catalytic activity.^4,5^ Based on the employed materials, nanozymes can be classified into materials mimicking the active centers of enzymes, enzyme functions, or organic–inorganic nanocomposites imitating entire enzyme structures.^6^ However, in each type of nanozyme, the addition of metals in various forms, such as oxides and ions, is essential for material preparation.^7,8^ The incorporation of metals can alter the properties of the nanomatrix, including its surface chemistry, charge distribution, and catalytic activity.^9^ Additionally, metals act as cofactors or modulators of the activity of natural enzymes. For example, the addition of divalent metal ions, such as manganese (Mn^2+^) or copper (Cu^2+^), can enhance the stability and activity of certain enzymes. These metal ions can coordinate with specific amino acid residues in the enzyme structure, providing structural support and promoting proper folding as they are typically present in metalloproteins.^10,11^ Therefore, the addition of metals (metal ions and metal oxides) can significantly enhance the nanozymatic activity of nanomaterials by mimicking the active sites of enzymes, especially in metalloproteins.^12^ Incorporating metals into nanomaterials can provide additional catalytic sites, promote electron transfer, and modulate redox activity properties, leading to increased catalytic efficiency.^13^ For example, adding metal ions such as Cu and Mn may promote the generation of reactive oxygen species or synergistic redox actions to mimic enzyme activities.^14^ Copper and manganese ions are two of many important metals in living organisms.^15^ They are present in natural enzymes from groups of hydrolases or oxidoreductases.^16,17^ Manganese is a metal that acts as an electron acceptor during intermediate formation in enzymatic catalysis reactions, and additionally, in natural enzymes, stronger oxidants can be processed by manganese-containing enzymes.^17^ Copper is needed for homeostasis of human organisms, and it can serve as a sensor of oxidative stress (Cu-dependent peptidase) in mitochondria and also take part in antioxidation processes.^18^ Therefore, using them in nanozymes is a way to follow naturally occurring catalytic systems. These metal-induced enzymatic actions have found applications in various fields of nanozyme usage, including environmental remediation, biosensing, and biomedical applications.^19^

These observations facilitate the rational design of nanomaterials exhibiting nanozymatic behavior, leading to improved catalytic efficiency and potential applications in various fields. However, to analyze and predict the behavior of nanomaterial systems, verification approaches based on fundamental nanozyme activities and characteristics can be applied. The aim of the study was to evaluate the effect of metal doping on the potential of multioxide nanomaterials to acquire nanozymatic activity. Three types of nanomaterials with diverse compositions containing oxide structures and varying surface characteristics were selected. Consequently, oxide nanomaterials and silicate–phosphate nanomaterials were obtained, which were then modified with manganese and copper. To determine whether a nanomaterial possesses nanozyme properties, assessing the levels of proteolytic activity and antioxidant activity in combination with physicochemical characterization techniques can be employed. It is assumed that proteolytic activity evaluates the nanomaterial’s ability to degrade proteins (suggesting a hydrolase mechanism of action), while antioxidant activity evaluates its ability to scavenge free radicals (suggesting an oxidoreductase-based mimicry). These enzymatic tests used as a screening tool for nanozyme activity, along with material characterization, provide valuable insights into the catalytic properties and potential applications of nanomaterials.

## 2 Methods

### 2.1 Materials

Copper(II) acetate [Cu(CH_3_COO)_2_·H_2_O] (Sigma-Aldrich), zinc(II) acetate [Zn(CH_3_COO)_2_·H_2_O] (Sigma-Aldrich), and manganese(II) acetate [Mn(CH_3_COO)_2_·H_2_O] (Sigma-Aldrich) were used as oxide nanoparticle precursors. Sodium metasilicate (Na_2_SiO_3_) (Sigma-Aldrich), hydrochloric acid (HCl) (Sigma-Aldrich), sodium hydrogen phosphate (Na_2_HPO_4_) (Sigma-Aldrich), potassium hydrogen phosphate (KH_2_PO_4_) (Sigma-Aldrich), calcium nitrate [Ca(NO_3_)_2_] (Sigma-Aldrich), and magnesium chloride (MgCl_2_) (Sigma-Aldrich) were used in phosphate nanomaterial precursors. Sodium hydroxide (NaOH) (Sigma-Aldrich) was used as a precipitating agent. The enzyme used as reference material was protease (Sigma-Aldrich). Materials used in activity tests and sorption were as follows: trichloroacetic acid (TCA) (Sigma-Aldrich), 6-hydroxy-2,5,7,8-tetramethyl-chromane-2-carboxylic acid (TROLOX) (Sigma-Aldrich), Folin–Ciocâlteu phenolic reagent (Sigma-Aldrich), casein (Sigma-Aldrich), and 2,2′-azino-bis(3-ethylbenzothiazoline-6-sulfonic acid (ABTS) (Sigma-Aldrich).

### 2.2 Synthesis of Metal Oxide Nanoparticles

To obtain metal oxide nanoparticles, a precipitation method and microwave-radiation-induced dehydration were employed. In the first stage, a zinc acetate dihydrate solution (C = 2M, V = 15 mL) was directly added to a Teflon container and precipitated with a sodium hydroxide solution (C = 4M, V = 15 mL) while simultaneously mixing using a Hielscher UP400 St ultrasonic processor for 2 min. Subsequently, the mixture with a total volume of 30 mL was transferred to a MAGNUM II microwave reactor, applying process parameters as follows: t = 15 min, T = 200 °C, and p = 25 bar. The material was filtered, washed, and dried at 70 °C for 24 h. In the second stage, the previously obtained ZnO was mixed with a manganese acetate solution in the appropriate molar ratio for 30 min in a Teflon container. Then, sodium hydroxide (C = 2M, V = 20 mL) was added dropwise to the container, which had a total volume of 40 mL, and the container was placed in the MAGNUM II microwave reactor to carry out the process for obtaining metal oxide nanomaterials. After a 10 min process, the material was poured and filtered using 0.1 μm nitrocellulose filters on a vacuum system, followed by drying at 70 °C. Upon drying, the material was ground in an agate mortar.

The materials were synthesized in molar ratios of Zn/Mn of 1:0.1 or 1:0.3 and Zn/Mn/Cu of 1:0.1:0.1, 1:0.1:0.3, 1:0.3:0.1, and 1:0.3:0.3, respectively, for binary or ternary oxide systems. Ternary oxide materials were obtained in the same manner, where after obtaining the zinc oxide nanomaterial, copper acetate was added in the appropriate molar ratio to the nanomaterial, followed by repeating the steps from stage II. The materials were labeled as ZnO-MnxOy-CuO with the corresponding manganese-to-copper ratio (e.g., ZnO-MnxOy-CuO 1:1, indicating a material containing manganese and copper additives in ratios of Zn/Mn/Cu of 1:0.1:0.1).

### 2.3 Synthesis of Metal-Modified Phosphate Glasses

The synthesis of glassy materials [SiO_2_–MgO–K_2_O–P_2_O_5_–CaO (FG) and SiO_2_–CaO–Na_2_O–P_2_O_5_ (BG)] involved preparing suspensions of metal hydroxides in appropriate molar ratios and conducting a microwave process to obtain metal oxides. The mol % composition of the FG material was as follows: 30% SiO_2_, 15% MgO, 10% K_2_O, 15% P_2_O_10_, and 30% CaO, and for the BG material, the composition was as follows: 45% SiO_2_, 24.5% CaO, 24.5% Na_2_O, and 6% P_2_O_10_.

In the first stage, a suspension of sodium metasilicate (1M, V = 20 mL) and hydrochloric acid (36%, V = 5.16 mL) was prepared and mixed for 30 min at room temperature. Then, mixture I was washed with water in a Büchner funnel. After washing and filtration, in the second stage, the suspension was mixed with the appropriate salts: sodium hydrogen phosphate (0.025M, V = 50 mL) and/or potassium hydrogen phosphate (0.01M, V = 10 mL) for 10 min, obtaining mixture II. Simultaneously, calcium nitrate (0.03M, V = 10 mL) was precipitated with sodium hydroxide (0.06M, V = 5 mL) and/or magnesium chloride (0.015M, V = 10 mL) with sodium hydroxide (0.03M, V = 10 mL) to hydroxides, which were added to mixture II and mixed for 10 min. In mixture III, metal salts (copper acetate or manganese acetate) were added in the appropriate molar ratio and mixed for 5 min. The molar ratios of additives were determined relative to the molar mass of the glassy material as 1:0.1 and 1:0.3. For the appropriate molar ratio, manganese acetate was added: C = 0.003M, V = 9.74 mL or C = 0.009M, V = 3.25 mL, and copper acetate: C = 0.003M, V = 8.54 mL or C = 0.009M, V = 25.63 mL.

The resulting mixture IV (containing the addition of one of the metals) was transferred to a Teflon vessel (approximately 60 mL) and subjected to a hydrothermal process in a microwave reactor under the following conditions: *t* = 20 min, *T* = 200 °C, and p = 40 bar. In this way, materials with different compositions of oxides and their percentage ratios were obtained.

The materials were labeled with the abbreviation of the name (BG or FG) and the symbol of the added metal (Mn or Cu) and a number (1 or 3), depending on the ratio of the metal used, e.g., BG-Mn-1, indicating a material containing glass with a composition of SiO_2_–CaO–Na_2_O–P_2_O_5_ with the addition of manganese in a ratio of 1:0.1 BG/Mn.

### 2.4 Physicochemical Characterization

The morphology of the obtained materials was examined using scanning electron microscopy (Hitachi TM 3000) for visualizing the particles and revealing their shape. The analysis of the crystallographic structure of nanoparticles was carried out using the X-ray diffraction (XRD) structural analysis method utilizing a Philips X’Pert Camera diffractometer with a PW 1752/00 CuKa monochromator in the 2θ angle range from 10 to 80°. Additionally, the crystallite size was calculated using the Scherrer equation for oxide materials
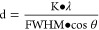


where d – average size of crystallites; fwhm – peak width at half of its height, proportional to the size of the crystallite; K – Scherrer’s constant; λ – X-ray wavelength; and θ – angle formed by radiation with the atomic plane.

Porous structure parameters [Brunauer–Emmett–Teller (BET) surface area, pore volume, and pore diameter] were determined using an ASAP 2020 physisorption analyzer (Micromeritics Instrument Co., USA) by the BET method based on low-temperature N_2_ sorption. Before measurement, the materials were degassed at 110 °C. The surface area was determined by the multipoint BET method using adsorption data in a relative pressure (p/p_0_) range of 0.01–1.

The X-ray photoelectron spectroscopy (XPS) measurement was performed for phosphate glasses for the determination of incorporated metal states in an ultrahigh vacuum (UHV, Prevac) chamber with a monochromatized Al Kα source (photon energy = 1486.6 eV) by VG Scienta XM 780. The photoelectrons were collected by an analyzer (Scienta R4000) with a pass energy of 200 eV (step 750 meV) and a range of 0–1210 eV.

### 2.5 Determination of Proteolytic Activity

To examine the potential enzymatic hydrolytic activity, methods determining the proteolytic activities of the tested materials were employed using casein as a substrate. During the hydrolysis of casein protein, reaction products were precipitated with TCA and subsequently, by a colorimetric method, determined using a Folin solution to measure the concentration of reaction products. The standard curve of the method was based on tyrosine, which is released during protein degradation.

For the tested sample (10 mg), a solution of casein (5 mL) was added, and the reaction was carried out for 30 min at 37 °C. Subsequently, the reaction was stopped with TCA (5 mL, 110 mM) and incubated for an additional 30 min. A filtrate (2 mL) was then collected, followed by determination using the Folin solution (2 mL), with the mixture incubated for 30 min. In the final step, the absorbance of the reaction filtrate with the Folin reagent (2 mL) was measured spectrophotometrically at 726 nm.

The standard curve was established within the range of 0–0.05–0.1–0.2–0.4–0.5 tyrosine concentration (μmol). From the equation of the curve, based on the measured absorbance (*y*), the amount of released tyrosine (*x*) was calculated
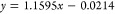

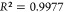


where *y* represents the measured absorbance and x represents the amount of released tyrosine (μmol).

The result was calculated by considering the volume of the reaction mixture (V_*t*_ = 11 mL) and time (*t* = 10 min), the volume of enzyme or sample (V = 1 mL), the volume after filtration (V_f_ = 2 mL), and the concentration of the sample or enzyme (C 10 mg/mL), obtaining the final result expressed as unit/mg. The following formula was utilized
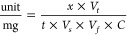


### 2.6 Determination of Antioxidant Activity

In the ABTS method, the absorbance of the ABTS + radical was measured spectrophotometrically using Trolox as a reference substance. The ABTS + radical is generated by 2,2′-azino-bis(3-ethylbenzothiazoline-6-sulfonic acid). This radical is a chemically stable chromophore compound with a wide pH range, is soluble in water, and exhibits strong light absorption in the range of 600–750 nm. Subsequently, antioxidants scavenge the ABTS + radical [2,2′-azino-bis(ethylbenzothiazoline-6-sulfonic acid)], leading to a decrease in absorbance, which is detected by the antioxidant–radical combination at various time points.^20^

The ABTS solution (6 mM) was prepared by dissolving potassium persulfate (2.45 mM) in water, and the solution was left in the dark for 16 h to age. A sample (0.1 mL of enzyme or 10 mg of nanomaterial) was added to 4 mL of the ABTS reagent, incubated for 10 min, and then measured at 734 nm. The calibration curve was based on the reaction of Trolox with the ABTS reagent, where the measured absorbance after the reaction corresponded to the amount of Trolox required for the reaction, expressed in μmol/mg. The calibration curve was prepared in the range of 0–40–80–120–160–200 μmol TROLOX, and the concentration was calculated from the following equation.
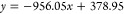

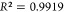
where *y* – absorbance and *x* – TROLOX concentration (μmol).

## 3 Results and Discussion

### 3.1 Synthesized Materials Characterization

**Figure 1:**
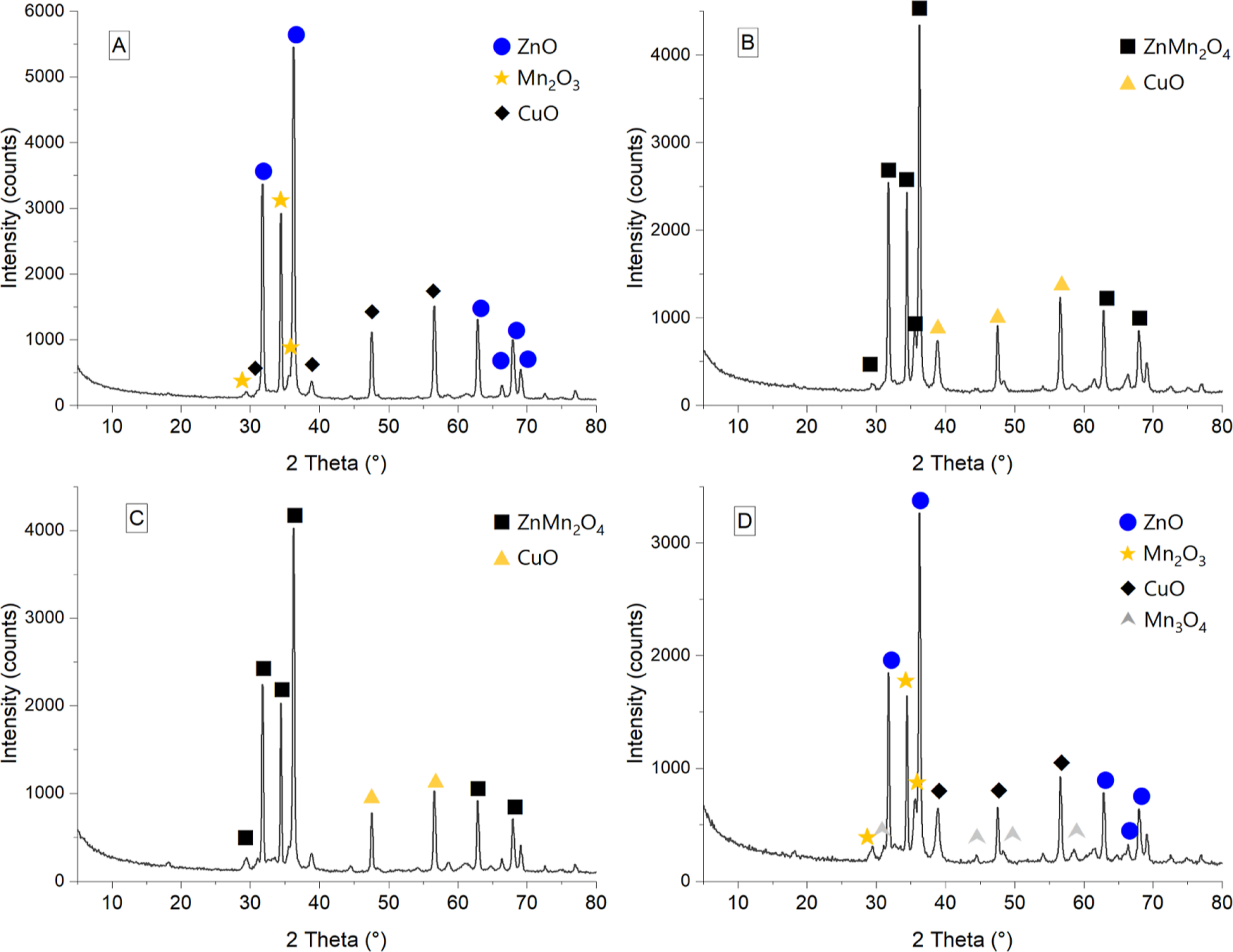
XRD patterns of material (A) ZnO-Mn_*x*_O_*y*_-CuO 1:1, (B) ZnO-Mn_*x*_O_*y*_-CuO 1:3, (C) ZnO-Mn_*x*_O_*y*_-CuO 3:1, and (D) ZnO-Mn_*x*_O_*y*_-CuO 3:3.

XRD studies of the materials confirmed the presence of oxide phases corresponding to each of the oxide nanomaterials (Figure 1). In all materials, the ZnO phase is present (31.78, 34.44, 36.28, 47.56, 56.62, 62.88, 66.38, 67.95, and 69.9°), consistent with the COD#2300116 database record.

In the copper-doped zinc oxide materials with copper content equal to manganese (ZnO-Mn_*x*_O_*y*_-CuO 1:1), the presence of the CuO phase (32.4, 38.6, 48.7, and 58.5°) and the Mn_2_O_3_ phase (29.4, 34.4, and 38.86°) was observed. Additionally, at the highest manganese and copper ratios (ZnO-Mn_*x*_O_*y*_-CuO 3:3), an additional Mn_3_O_4_ phase (31.11, 36.27, 44.5, 48.33, and 58.59°) appeared in the material. These phases correspond to records with the respective COD#7212242, COD#1514235, and COD#1011262 database numbers.

In materials with a mixed ratio of manganese and copper (Mn/Cu 3:1 or 1:3), phases corresponding to manganese at a mixed oxidation state of Mn^+III/+IV^ in the ZnMn_2_O_4_ structure (29.46, 32.86, 34.4, 36.28, 38.88, 61.00, and 68.0°) and the CuO phase (38.6, 48.7, and 58.5°) were identified. These phases are reflected in the crystallographic data, COD#2300116 and COD#9016105, respectively.

The crystal sizes were calculated based on the XRD data, and the average crystal sizes of the phases are presented in Table 1.

**Table 1:** Nanomaterial Phase Calculated Size in nm Based on XRD Results for Oxide Nanomaterials

material	size [nm]	phase
ZnO-MnxOy-CuO 1:1	46.20	ZnO
	27.27	Mn +II/+III
	15.41	CuO
ZnO-MnxOy-CuO 1:3	46.20	ZnO
	24.08	Mn +III/+IV
	15.17	Cu_2_O
ZnO-MnxOy-CuO 3:1	47.62	ZnO
	11.52	Mn +III/+IV
	29.85	Cu_2_O
ZnO-MnxOy-CuO 3:3	41.64	ZnO
	13.37	Mn +II/+III
	15.78	Mn +III/+IV
	12.96	CuO

The XRD results obtained for the ZnO phase align with those presented by Raha et al.^21^ The researchers synthesized a nanomaterial system CuO/Mn_3_O_4_/ZnO using a precipitation method, initially obtaining copper oxide, followed by zinc oxide and manganese oxide (+II/+III). The material was subjected to autoclaving to facilitate the reaction, followed by additional calcination. Additionally, the researchers characterized individual phases of the system, where the ZnO phase size was reported as 20, 10 nm for the CuO phase, and 14 nm for the Mn_3_O_4_ phase.^21^ The sizes of the individual nanomaterial phases reported by Raha et al. are consistent with the crystal sizes obtained in this study, as confirmed by the diffraction patterns for these phases.

The spinel phase ZnMn_2_O_4_ can be found in the XRD results presented by Islam^22^ and Sharma,^23^ focusing on the utilization of ZnMn_2_O_4_ as batteries and energy carriers. In Sharma‘s study,^23^ the material obtained by the researchers was also synthesized using a precipitation method, but the obtained sizes exceeded 30 μm. The formation of the ZnMn_2_O_4_ phase can be rationalized by the findings of Wang et al.,^24^ where the researchers investigated the influence of increasing Cu content on the formation of spinel phases in CuMnOx catalysts. According to their reports, Cu structures tend to form oxides or deposit on Mn surfaces rather than form spinel phases with Mn unless the addition of Cu is increased. Due to the increased addition of metals relative to the overall material, it is possible for a change in the oxidation state of Mn to occur in the nanomaterials, although the association with copper may not be favored.

### 3.2 X-ray Photoelectron Spectroscopy

**Figure 2:**
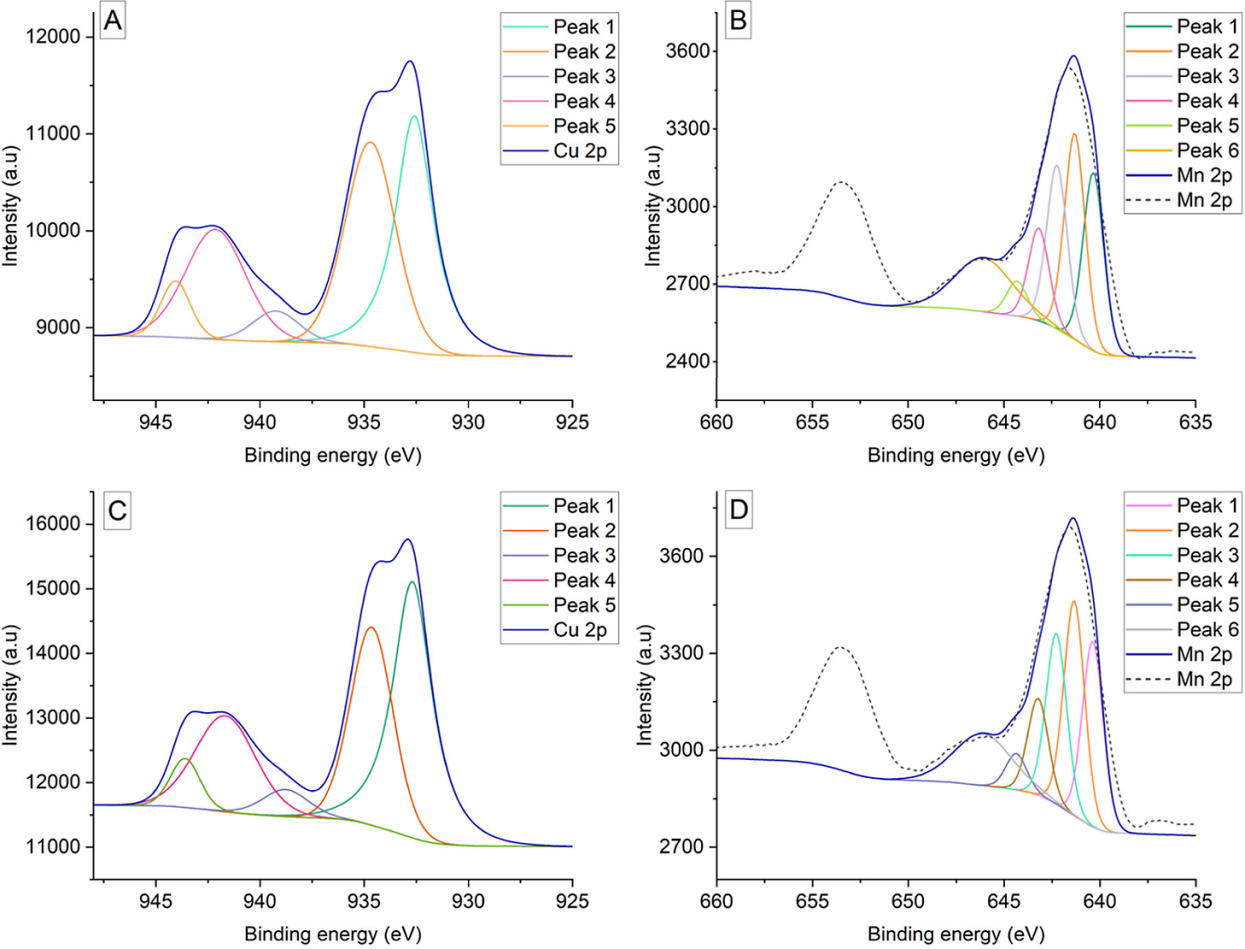
XPS results for copper and manganese in materials (A) BG-Cu-3, (B) BG-Mn-3, (C) FG-Cu-3, and (D) FG-Mn-3. Dashed lines in (B and D) represent the original smoothed data.

For the glassy materials, XPS analysis was conducted to identify the oxidation states of the metals added during synthesis. All XPS spectra for metals are shown in Figure 2, and peak energy matches after deconvolution are included in Table 2. According to the obtained results, bands for Mn 2p_1/2_ and Mn 2p_3/2_ with visible maxima at 653 and 640 eV, respectively, are present in both types of materials (BG-Mn-3 and FG-Mn-3, dashed line), which shows the divalent nature of manganese dopant.^25^ Detailed deconvoluted data from the first band (Table 2) show peak positions, peak area percentages, and pass energy values according to Biesinger,^26^ where 5 major peaks between positions from about 640 to 644 eV are highlighted, with transition energies of 1.26 eV (BG-Mn-3) or 1.27 eV (FG-Mn-3) representing the Mn (II) oxidation state. Characteristic satellite peak 6 at 646.09 or 646.12 eV (accordingly) is present in both samples with 3.5 eV fwhm pass energy; this is also in accordance with literature data.^27^ According to Chen D. and their group,^25^ prepared MnO nanofibers showed similar XPS data to materials in this study with two spectra bands for Mn 2p_1/2_ (653.6 eV) and Mn 2p_3/2_ (641.5 eV) and a satellite peak at 644.7 eV, which suggests the presence of mainly MnO form in materials of this study. Consistent with the studies of Barrioni,^28^ who analyzed addition of manganese (0–2,5–5%) to calcium-phosphate glass with 58S composition, the data for the glassy material obtained by the research group align with those obtained in this study.

**Table 2:** Spectral Fitting Data for Analyzed Samples: Binding Energy (eV), FWHM Value (eV) for Each Pass Energy, and Percentage of Total Area (%)

sample identifier	name	oxidation state	binding energy (eV)	fwhm	%
BG-Cu-3	peak 1	Cu (0)	932.59	2.15	34.4
	peak 2	Cu (II) hydroxide	934.70	2.86	33.9
	peak 3		939,24	2.60	4.6
	peak 4		942.14	3.40	21.7
	peak 5		944.06	1.66	5.4
BG-Mn-3	peak 1	Mn (II)	640.35	1.26	21.6
	peak 2		641.32	1.26	25.0
	peak 3		642.25	1.26	19.9
	peak 4		643.20	1.26	11.2
	peak 5		644.34	1.26	4.2
	peak 6		646.09	3.51	18.1
FG-Cu-3	peak 1	Cu (0)	932.69	2.23	41.0
	peak 2	Cu (II) hydroxide	934.64	2.41	29.5
	peak 3		938.78	2.61	4.3
	peak 4		941.68	3.41	20.2
	peak 5		943.60	1.67	5.0
FG-Mn-3	peak 1	Mn (II)	640.38	1.27	21.9
	peak 2		641.35	1.27	25.4
	peak 3		642.28	1.27	20.2
	peak 4		643.23	1.27	11.4
	peak 5		644.37	1.27	4.3
	peak 6		646.12	3.50	16.9

**Figure 3:**
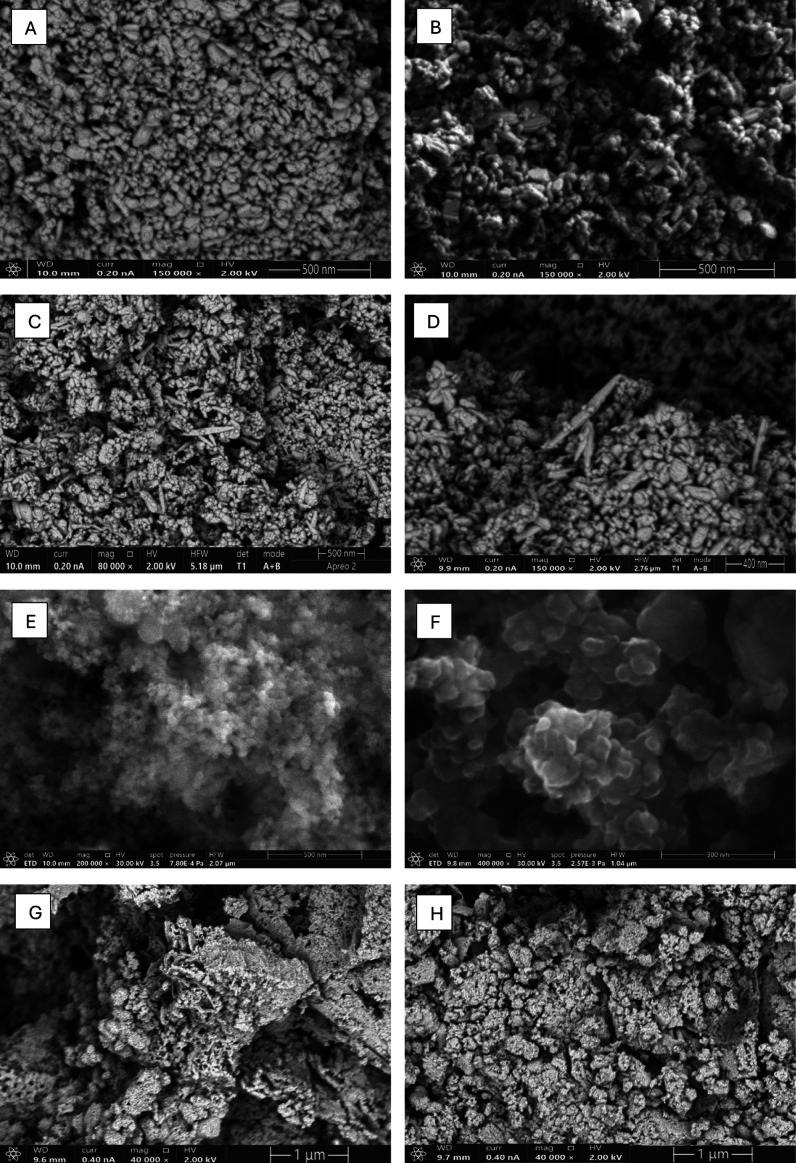
SEM microphotographs for (A) ZnO-Mn_*x*_O_*y*_-CuO 1:1, (B) ZnO-Mn_*x*_O_*y*_-CuO 1:3, (C) ZnO-Mn_*x*_O_*y*_-CuO 3:1, (D) ZnO-Mn_*x*_O_*y*_-CuO 3:3, (E) BG-Mn-3, (F) BG-Cu-3, (G) FG-Mn-3, and (H) FG-Cu-3.

In the case of adding copper to the glassy materials, at 932 eV, a characteristic double-peak band can be seen, and a closely related band at 942 eV is visible in the Cu 2p XPS spectra. Similar to the findings of Chitra,^29^ where researchers analyzed the presence of copper additives in calcium–phosphate bioglass, a Cu 2p band was visible. Additionally, the research described the presence of Cu(OH)_2_, which is consistent with this study. According to Biesinger,^30^ deconvoluted peaks for Cu 2p are presented in Table 2, where the positions of peak 1 are related to metallic Cu and peaks 2–6 are related to Cu^2+^ found in hydroxide. These findings are also confirmed by the research of Christophliemk et al.,^31^ where a similar doubled band at 932 eV is described. Additionally, the research indicates that the Cu 2p spectra for the copper hydroxide peak at 932 eV are characteristic of this compound. Similar findings were stated in the work of Liu et al.^32^

### 3.3 Scanning Electron Microscopy

First, SEM micrographs (Figure 3) revealed that the shape of the nanomaterials was predominantly spherical, with nanorods present. Similar morphologies were noted in the studies by Mohamed et al.^33^ for ZnO, Mn3O4, and CuO nanoparticles—the addition of manganese and copper resulted in the formation of more spherical particles with concurrently existing nanorods. In the case of the BG glassy material, SEM micrographs showed spherical nanomaterials and the presence of a spongy, granular structure with visible material pores. This spongy nature may enhance the material’s bioactivity by providing additional surface area for ion release and cell attachment.^34^ Additionally, SEM micrographs provided information regarding the approximate size of the materials. It was determined that the particles of phosphate glasses had an average size of about 100 nm, while oxide nanoparticles had an average size of about 50 nm. Similar material sizes were observed in the studies by Fayad et al.,^35^ where the influence of metals on the bioactivity of glassy materials was compared, and for oxide nanoparticles in the studies by Raha et al.^21^

### 3.4 BET Method

**Figure 4:**
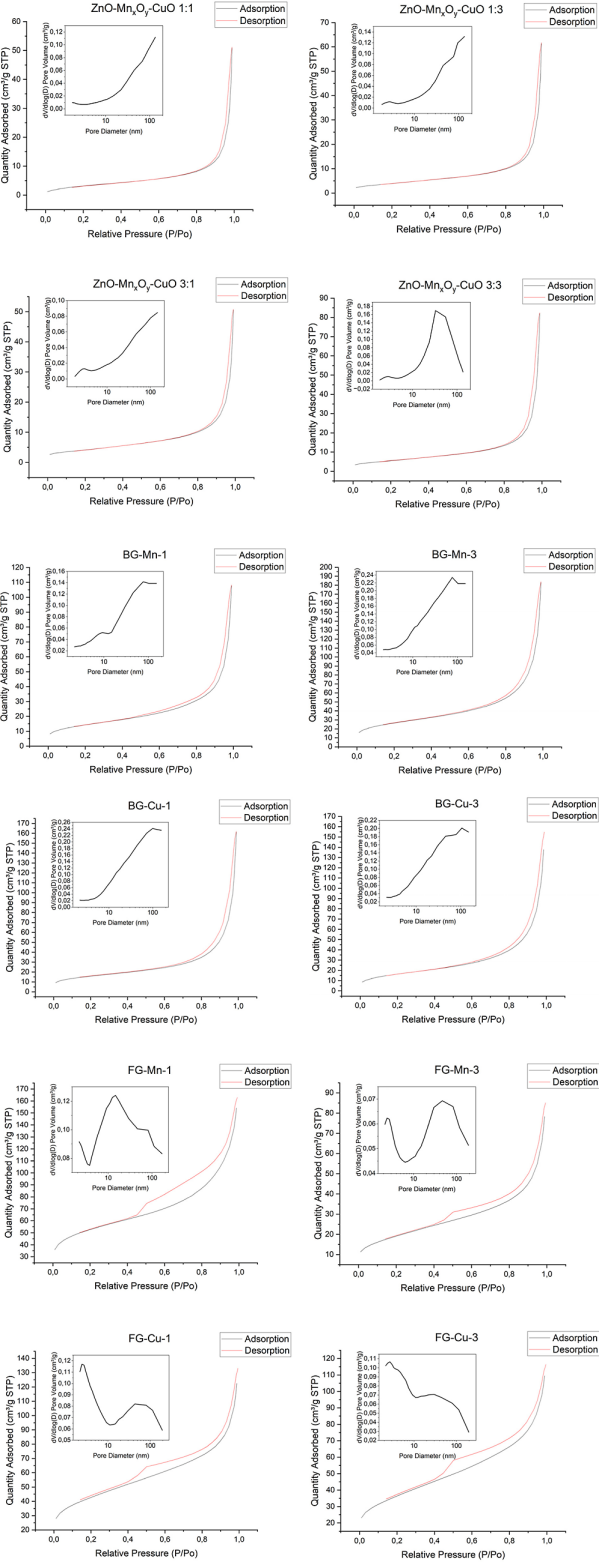
Adsorption–desorption isotherms for materials and pore volume distribution for the obtained materials.

The nitrogen adsorption–desorption isotherms for the samples and the pore size distributions are presented in Figure 4. The curves for the oxide materials can be characterized as Type II isotherms, indicative of macroporous structures,^36^ which is further confirmed by the pore size distributions (Figure 4) – the materials exhibit pores with sizes close to 100 nm. Similarly, the curves for the BG materials can be characterized by pore size distributions, indicating the presence of macropores in the range of 90–100 nm. The isotherms for the FG glassy materials can be characterized as Type III hysteresis loops according to the IUPAC classification, characteristic of mesoporous materials with slit-like pores.^37^ Such isotherms are typically observed for hierarchical porous materials with a broad range of pore size distributions, as evidenced by the pore size distribution plots (Figure 4), where micropores and mesopores around 20 nm and between 40 and 80 nm are observed. They exhibit increased adsorption at high pressures approaching the saturation vapor pressure.^38^ Additionally, from the pore distributions presented in Figure 4, it can be observed that only the FG glassy materials exhibit the presence of both micropores and mesopores, while the ZnO-MnxOy-CuO 3:3 oxide material is the only oxide material showing mainly mesopores, which is attributed to the packing of the oxide structure and the varying sizes of manganese and copper ions.

Low-temperature nitrogen adsorption analysis was employed to determine the surface porosity of the synthesized materials: surface area (SBET), total pore volume (V_*t*_), and average pore size (nm). The data for each characterized material are listed in Table 3. The highest surface area values were achieved by the glassy materials, particularly the FG material. Changes in material composition did not significantly affect the total pore volume; however, differences in pore size were observed among the oxide materials and each type of glassy material. Higher additions of both manganese and copper led to an increase in the surface area of the oxide and BG materials, while in the FG glasses, a higher metal addition resulted in a decrease in surface area (in the case of manganese addition by half). The values obtained for the oxide nanomaterials are consistent with those reported in the literature—in the study by Yadav, the total surface areas for ZnO and CuO nanomaterials and ZnO/CuO were 18.128, 16.653, and 19.580 m^2^/g, respectively.^39^

**Table 3:** Pore Volume, Size, and BET Values for the Obtained Nanomaterials

material	SBET (m^2^/g)	total pore volume Vt (cm^3^/g)	Av. pore width (nm)
ZnO-MnxOy-CuO 1:1	12.5	0.0792	25.4
ZnO-MnxOy-CuO 1:3	14.6	0.0955	26.1
ZnO-MnxOy-CuO 3:1	14.9	0.0786	21.1
ZnO-MnxOy-CuO 3:3	19.7	0.1275	25.9
BG-Mn-1	50.9	0.1675	13.2
BG-Mn-3	93.9	0.2846	12.1
BG-Cu-1	56.3	0.2505	17.8
BG-Cu-3	59.8	0.2396	16.0
FG-Mn-1	187.9	0.2522	5.4
FG-Mn-3	69.2	0.1316	7.6
FG-Cu-1	149.8	0.2058	5.5
FG-Cu-3	129.5	0.1800	5.6

### 3.5 Proteolysis and Antioxidant Activity

**Figure 5:**
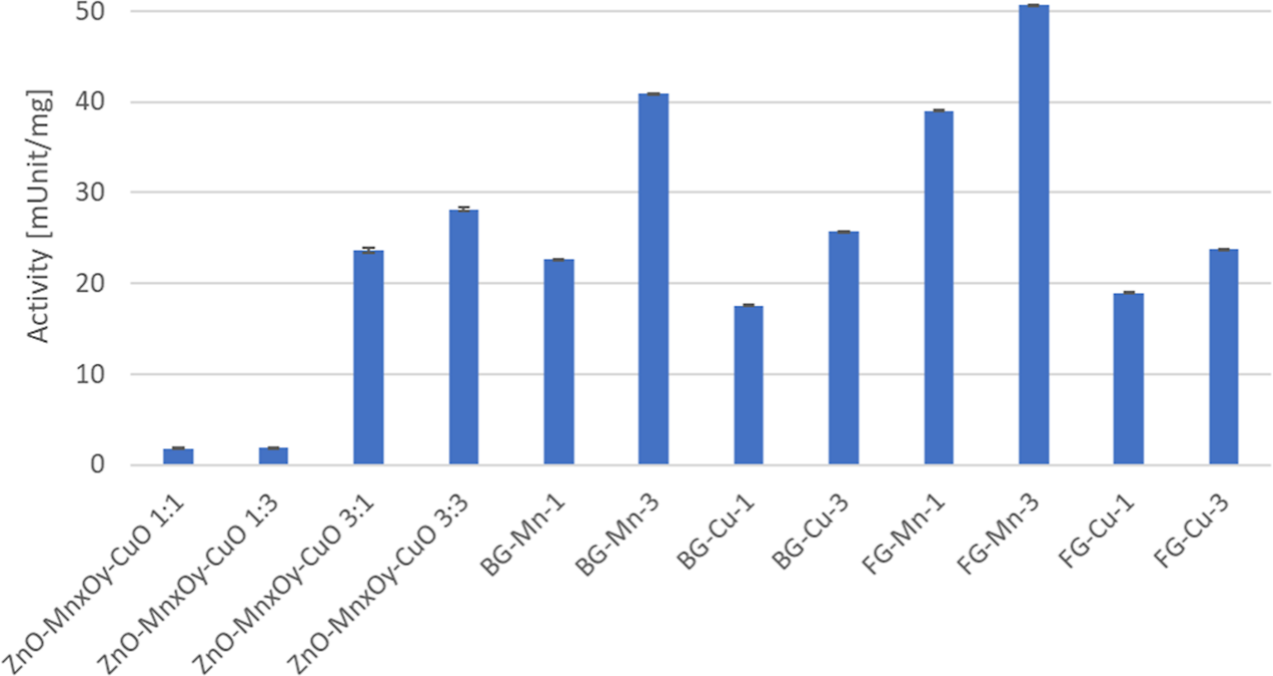
Proteolytic activity measured by casein decomposition in mUnit/mg of protease activity.

The investigated materials were subjected to enzymatic activity tests to assess their potential nanozymatic activity in the cases of casein degradation and ABTS radical reduction. In Figure 5, it can be observed that for each type of material, an increase in manganese content in the material enhanced proteolytic activity (even up to twice for the BG-Mn material—changing from 20 to 40 mUnit/mg). The activities of glassy materials containing copper (around 17–23 mUnit/mg) were lower than those of materials with manganese (around 25–50 mUnit/mg), although their activity increased by nearly 40% after copper addition. The lowest protein degradation activities were exhibited by oxide materials with lower manganese contents. According to Chen’s study,^40^ the investigated Mn_2_O_3_ and Mn_3_O_4_ oxide materials showed the highest oxidative activity, similar to enzymes from the oxidase or peroxidase groups, respectively. These structures are also present in the investigated oxide materials ZnO-MnxOy-CuO 3:1 and ZnO-MnxOy-CuO 3:3, indicating that materials with increased manganese content exhibit higher activity toward protein degradation.

**Figure 6:**
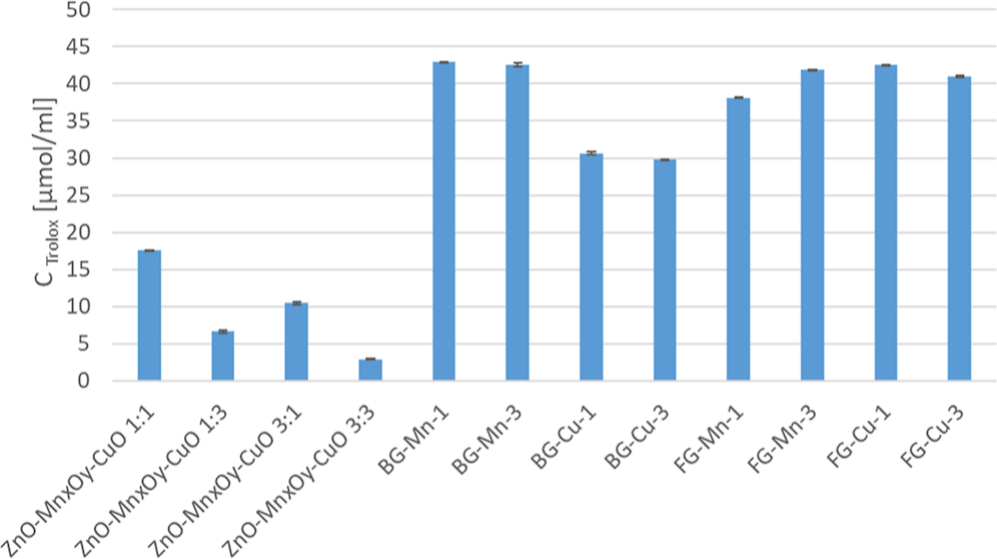
Antiradical activity of the synthesized nanomaterials in μmol/mL as the TROLOX concentration.

Furthermore, the antioxidant activity of the obtained materials was investigated (Figure 6). Glassy materials exhibited higher activities (ranging between 30 and 45 μmol/mL) compared to those of oxide materials (5–15 μmol/mL). According to Xi et al.,^41^ the nanozymatic activity of materials containing CuO or Cu^2+^ differs in mechanism and affects antimicrobial activity. Therefore, it can be inferred that copper in the hydroxide form present in glassy materials is more active than copper in the oxide form. Although antioxidant studies on glasses can be found in the literature and confirm that the addition of manganese affects the reducing activity,^42^ they do not allow for a comparison of the reducing activity in terms of the oxidoreductase mechanism. Similarly, studies by Neethidevan et al.^43^ regarding the use of CuO as an additive to enhance the antioxidant activity of plant extract confirm that the additive enhances the material’s activity against radicals; however, they do not provide a direct reference.

The noticeable impact of metal addition on proteolytic activity is observed, which increases with the concentration of the metal in the material. Simultaneously, higher proteolytic activity correlates inversely with antioxidant activity. This finding is justified by the oxidation states of the added metals, which form systems capable of redox reactions. In studies conducted by Qian^44^ on CuO/MnO_2_ catalysts with varying amounts of CuO addition for catalyzing CO oxidation, it was demonstrated that the material’s surface area (SBET) had no effect on the activity of the investigated materials in catalysis. Therefore, materials containing multivalence structures will exhibit higher proteolytic activity, indicating the acquisition of nanozymatic activity.^45^ Similar to the findings of Dey et al.,^46^ where CuMnO_*x*_ catalysts were investigated, it was shown that the activity of the catalyst was influenced by the availability of Cu–Mn structures with mixed oxidation states, allowing oxidation and reduction reactions between metals in the + II and + III oxidation states to occur. Additionally, there is a noticeable strong influence of manganese addition on the increase in protein degradation activity and antioxidant properties, which is reflected, among others, in the studies by Yue et al.,^47^ where the effect of metal additives on the redox activity of CeO_2_-based nanozymes was examined—the research determined that manganese addition had the strongest impact on the redox activity of the obtained nanozymes. For example, a material with high proteolytic activity and antioxidant action may exhibit the activities of enzymes from the hydrolase group, e.g., BG-Mn-3. Thus, the dominant factor in assessing the utility of a nanomaterial as a nanozyme will be the combination of these two characteristics, where proteolytic activity will be a crucial factor before the decision on enzyme immobilization.

## 4 Conclusions

In the presented study, nanooxide and glassy materials were characterized to determine whether the addition of metal as well as its form or degree of oxidation could influence the acquisition of nanozymatic properties. The obtained materials were tested for proteolytic activity (protein hydrolysis) and antioxidant activity. Verification of activity was analyzed along with structural studies of the materials to determine the validity of the hypothesis. As described above, although the large surface area of glassy materials and their porosity may favor increased nanozymatic activity, a significant factor in determining the activity of a potential nanozyme is the degree of oxidation of metals and the possibility of consecutive oxidation–reduction reactions. This finding is supported by the case of oxide materials, especially ZnO-Mn_*x*_O_*y*_-CuO 3:3, where two phases of Mn with different degrees of oxidation and the CuO phase are present—this material showed low antioxidation and high proteolytic activity, confirming the above conclusions. According to the cited studies above, it is evident that the nanomaterial system with Mn^2+^ will be the most active, which is confirmed in this study and is reflected in the results of both activity tests for glassy materials. Additionally, knowledge of the physicochemical characteristics of the material and the comparison of the results of proteolytic breakdown activity and antioxidant activity against the ABTS radical indicate which materials may constitute active nanozymes. High proteolytic activity may suggest that the material is capable of mimicking enzymatic reactions, especially hydrolytic ones, such as enzymes from the hydrolase or peroxidase groups. Similarly, high antioxidant activity will affect the rapid breakdown of radicals, and thus the material may exhibit activity like enzymes from the oxidoreductase group. Therefore, the selection of appropriate metals or their oxides, depending on the desired type of reaction^40^ and the combination of research with the verification of the biological activities of nanomaterials, may provide a solution for the initial characterization of a nanozyme, as demonstrated through the analysis of the oxidation states and activity of the obtained materials.
